# Supplementary material for: Household factors and the risk of severe COVID-like illness early in the U.S. pandemic
Source: PLoS One. 2022 Jul 21;17(7):e0271786. doi: 10.1371/journal.pone.0271786 (PMC9302833; doi:10.1371/journal.pone.0271786)
Supplement: S1 Table — (PDF) [file pone.0271786.s001.pdf]

S1 Table: Select socio-demographic, health and behavior characteristics among C3 participants with children in household and who completed first version of assessment (N=5348)

	Presence of Children in Household			
	**Total**	**Yes**	**No**	**p-value**
	**N (%)**	**n (%)**	**n (%)**	
**Total**	5348	1275 (23.8%)	4073 (76.2%)	
**Socio-demographic**				
**Age group (years)**				
**18-49**	3067 (57.4%)	1029 (80.7%)	2038 (50.0%)	< 0.0001
**50-59**	851 (15.9%)	159 (12.5%)	692 (17.0%)	
**60+**	1430 (26.7%)	87 (6.8%)	1343 (33.0%)	
**Gender**				
**Male**	2558 (47.8%)	478 (37.5%)	2080 (51.1%)	< 0.0001
**Female**	2636 (49.3%)	772 (60.6%)	1864 (45.8%)	
**Gender non-binary**	154 (2.9%)	25 (2.0%)	129 (3.2%)	
**Race/ethnicity**				
**Hispanic**	791 (14.8%)	372 (29.2%)	419 (10.3%)	< 0.0001
**White non-Hispanic**	3574 (66.8%)	649 (50.0%)	2925 (71.8%)	
**Black non-Hispanic**	516 (9.7%)	141 (11.1%)	375 (9.2%)	
**Asian/Pacific Islander**	249 (4.7%)	62 (4.9%)	187 (4.6%)	
**Other**	218 (4.1%)	51 (4.0%)	167 (4.1%)	
**Annual household income level**				
**< $50,000**	2678 (50.1%)	594 (46.6%)	2084 (51.2%)	< 0.0001
**$50,000- $99,000**	1203 (22.5%)	224 (17.6%)	979 (24.0%)	
**≥ $100,000**	1101 (20.6%)	333 (26.1%)	768 (18.9%)	
**Not reported**	366 (6.8%)	124 (9.7%)	242 (5.4%)	
**Health and behaviors**				
**Essential worker**				
**Yes**	1286 (24.1%)	410 (32.2%)	876 (21.5%)	< 0.0001
**No**	4062 (76.0%)	865 (67.8%)	3197 (78.5%)	
**Reported having comorbidities**				
**Yes**	1860 (34.8%)	426 (33.4%)	1434 (35.2%)	0.2400
**No**	3488 (65.2%)	849 (66.6%)	2639 (64.8%)	
**Had close contact with suspected/confirmed case**				
**Yes**	801 (15.0%)	300 (23.5%)	501 (12.3%)	< 0.0001
**No**	4547 (85.0%)	975 (76.5%)	3572 (87.7%)	
**Household Factors**				
**Property type**				
**Multi-unit property**	1978 (37.0%)	400 (31.4%)	1578 (38.7%)	< 0.0001
**Single-unit property**	3008 (56.3%)	765 (60.0%)	2243 (55.1%)	
**Other**	362 (6.8%)	110 (8.6%)	252 (6.2%)	
**Number of persons living in household**				
**1**	1477 (27.6%)	0	1477 (36.3%)	< 0.0001
**2-3**	2475 (46.3%)	316 (24.8%)	2159 (53.0%)	
**4+**	1396 (26.1%)	959 (75.2%)	437 (10.7%)	
